# Utilising Family-Based Designs for Detecting Rare Variant Disease Associations

**DOI:** 10.1111/ahg.12051

**Published:** 2014-01-01

**Authors:** Mark D Preston, Frank Dudbridge

**Affiliations:** 1 London School of Hygiene and Tropical MedicineKeppel Street, London, WC1E 7HT, UK

**Keywords:** Rare variants, family-based association, TDT, missing heritability

## Abstract

Rare genetic variants are thought to be important components in the causality of many diseases but discovering these associations is challenging. We demonstrate how best to use family-based designs to improve the power to detect rare variant disease associations. We show that using genetic data from enriched families (those pedigrees with greater than one affected member) increases the power and sensitivity of existing case–control rare variant tests. However, we show that transmission- (or within-family-) based tests do not benefit from this enrichment. This means that, in studies where a limited amount of genotyping is available, choosing a single case from each of many pedigrees has greater power than selecting multiple cases from fewer pedigrees. Finally, we show how a pseudo-case–control design allows a greater range of statistical tests to be applied to family data.

## Introduction

We are interested in the effect on the power of statistical tests for rare variants when using family-based genetic data. The main question that we address in this paper is: *how best to use family-based designs to detect rare variant disease associations?* To answer this question, we must investigate two related aspects of the problem: which designs are most efficient and which statistical test(s) have the highest power to detect associations. Our results will enable us to inform future study design and how best to analyse existing family-based genetic data.

Genome-wide association studies (GWAS) are powerful tools for locating common genetic variants that affect heritable traits, such as disease susceptibility, but these variants do not explain the majority of the heritability of most traits. The remaining heritability is likely to arise from genetic interactions or rare genetic mutations (Maher, 2008; Gibson, 2012) and it is the detection of rare variants that we are concerned within this study.

Most GWAS are based on unrelated subjects, particularly case–control studies in which allele frequencies in the affected subjects are compared to those in the unaffected. An alternative is to utilise information from the family structure of the samples and measure the difference in transmission and nontransmission frequencies of minor alleles to the affected subjects from their parents (Spielman et al., 1993; Lake et al., 2000). Family-based analyses have a number of advantages including controlling for population stratification and offering allele enrichment (Ott et al., 2011). Between different strata (or sub-populations) there is the possibility of different allele frequencies giving rise to false positive disease associations. Testing the allele transmission controls for this under the assumption that transmissions of either allele from each heterozygous single nucleotide variant in both parents are independent. Furthermore, alleles that increase disease susceptibility are more prevalent in families that have a history of affection (Risch & Teng, 1998) and this, in turn, offers more power to statistical tests through so called enrichment (Antoniou & Easton, 2003).

Low allele frequencies of rare variants (<1%) cause single nucleotide polymorphism (SNP) association analyses, such as those used in GWAS, to be underpowered to detect their effects (Laird & Lange, 2006). This has led to the development of rare variant specific tests. These include tests that aggregate a set of variants into one “super” variant to increase allele frequency and hence the power of tests to detect associations. Early examples include the cohort allelic sum (Morgenthaler & Thilly, 2007) and the combined multivariate and collapsing (CMC, Li & Leal, 2008) tests. However, collapsing multiple variants into one introduces new problems. When the set of collapsed variants includes nonassociated variants then signal is weakened through increased noise. If causal variants with positive and negative associations are pooled together then they can (partially) cancel each other out, degrading the signal further. Research has since gone in two directions: how to improve pool selection (Zhang et al., 2010) and the invention of tests to overcome these limitations including the sequence kernel association (SKAT; Wu et al. 2011), kernel-based adaptive clustering (KBAC; Lui & Leal, 2010) and C-alpha (Neale et al., 2011) tests.

These rare variant tests aim for data enrichment through pooling mechanisms and are not designed for, and therefore do not take advantage of, the enrichment of rare variants to be found in families. The problem investigated within is how best to combine rare variant tests and the enrichment of family-based data for improving the detection of rare variant disease association. We will restrict attention to disease affection status as a binary trait, deferring consideration of quantitative traits to future work.

Although the potential of family data has been recognised for some time, there has been relatively little work on adapting methods for case–control data to family designs. Zhu & Xiong (2012) presented adaptations of Hotellings T^2^, the CMC test of Li & Leal (2008), and two tests based on principal component analysis of multiple genotypes, and for dominant models only, showed improved power compared to case–control designs of comparable size. De et al. (2013) adapted the family based association tests to combined rare variants, finding similar power to comparable case–control studies. Fang et al. (2012) used between-family information to derive weights for each rare variant in a combined within-family test. In contrast to those studies, we take as our point of departure the best performing multivariate tests for case–control data (Basu & Pan, 2011) and also consider the relative power of different family designs. We do not consider the assignment of weights to each rare variant as this can be treated separately to the general form of the test statistic chosen.

There are two paths one can choose between when analysing genetic data from families for rare variant association with disease. The first is to utilise pedigree structure and perform analysis on statistics derived from allelic transmissions and pedigree structure. The second is to transform or replace parts of the family-based data and apply specific rare variant case–control statistical tests. In this paper we will investigate both routes.

We begin by describing five new family-based statistical tests, derived from well-known case–control score tests and based on the allelic transmissions of the parents to an affected offspring. We use these tests, with a standard battery of statistical tests for both rare and common variants. In the alternative route, we examine two ways to use case–control tests with the family data. The first method is to create pseudo-controls from the untransmitted alleles of the parents of each affected offspring (Cordell et al., 2004) (pseudo-case–control, PCC) and the second is to retain all of the cases and include new unrelated controls (unrelated-case–control, UCC).

To determine the characteristics of the proposed tests we apply them to data simulated in a multitude of different scenarios. For each scenario we perform a set of replications to determine power and error levels of each test. In the Materials and Methods section we define all of the statistical tests that are used, including the new family-based tests, the techniques used to simulate the genetic data and the scenarios that we use to generate the results. We present our main results for the rare variant analysis in the Results with additional results to be found in the Supporting Information. Finally, in the Discussion section, we put our results on rare variant detection in context, for consideration in the design of future experiments as well as the applicability to existing family-based data.

## Materials and Methods

In this section, we define the statistical tests and scenarios that we will use to investigate how best to leverage family-based genetic data for rare variant disease association analysis. This includes defining five new score-based tests to take advantage of family-based genetic data and two transformations that can be applied to family-based data to create case–control data. We define a set of simulated scenarios to which we apply all of these statistical tests in order to elucidate the most appropriate family-based and/or rare variant analyses to use under a variety of conditions.

We will assume a known dichotomous (binary) disease status and measured genotypes for a set of variants for each subject. We assume that no data are missing and that the variants may occur anywhere in the autosomes. We do not consider covariates although some of the tests have the capability to include them. The question of disease association we are answering with respect to these data is:

does having a minor allele at any of the variants confer a change in an individual’s disease susceptibility?

To answer this question all of the tests employ the same null hypothesis, namely that none of the SNPs have any effect on the individual’s susceptibility to the disease. This leads to the alternative hypothesis that one, or more, of the SNPs in the group do have an effect on disease susceptibility. The definition of the alternative hypothesis implicitly includes two important points: we do not assume a deleterious effect and we are not testing individual variants for association but the group as whole. This then puts us in a position to compare single SNP analyses with multiple test correction with the grouped analyses used in current rare variant tests.

### New Family-Based Score Tests

We have devised five new family-based score statistics derived from the well-known score statistics. These new statistics are calculated from the heterozygous parental transmission frequencies to affected offspring, in a similar manner as the transmission disequilibrium test (TDT). We consider independent nuclear families consisting of both parents and a number of full siblings; here we restrict attention to parent-child trios and parent–sib-pair families. We assume that we are not working under a prior hypothesis of linkage so that we may treat transmissions to each sib as independent, and therefore treat sib-pair families as two independent trios.

We define *n* to be the number of trios in the data and *m* to be the number of variants then we define two 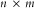 matrices 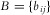 and 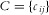 to contain the transmission information from parents in each each of the trios. From the *i*^th^ trio, we let 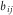 be the count of minor alleles transmitted to the affected offspring from the parent(s) that are heterozygous at the *j*th variant. Similarly, we define 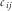 as the count of the major alleles transmitted under the same conditions. Each value in *B* and *C* takes the values 0, 1 or 2. The transmission disequilibrium found across the trios over all the variants is measured by 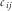.

Combining *B* and *C*, we calculate the family-based transmission disequilibrium as 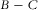. From this, we calculate a family-based score vector *U* and its covariance matrix *V* as

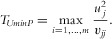
where 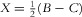 with 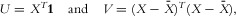, i.e. each column being filled with the column average from *X*.

The first score statistic is the maximum single SNP test, termed UminP by Pan (2009). The UminP statistic is given by

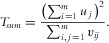
It has an asymptotic null distribution given by a combination of χ^2^ distributions (Conneely & Boehkne, 2007) and is equivalent to testing the significance of the minimum *P*-value given by the single SNP association/TDT analysis of the set of SNPs.

The next three statistics use different estimators of the variance of 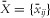. The score statistic is given by

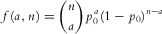
and the sum of squared score (SSU) and weighted sum of squared score statistics (SSUw; Pan, 2009) are

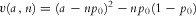
The score statistic (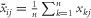) utilises the full covariance matrix to generate a statistic that takes account of the (estimated) linkage disequilibrium structure. The SSU and SSUw are simplified forms of the score statistic that take into account less information about the correlation between the variants. The asymptotic null distribution for 
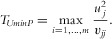
 is χ^2^ with *m* degrees of freedom and for 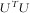 and 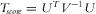 the distributions under the null hypothesis are combinations of scaled and shifted χ^2^ distributions (Pan, 2009).

The last test we use that is derived from *U* and *V* is the called the sum test, with statistic

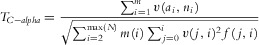
This final score statistic assumes that all variants have an effect on disease susceptibility, moreover that the effect of a minor allele occurring at each variant is the same, i.e. in the logistic model 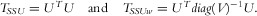 for all *i*. This has the advantage of reducing the number of degrees of freedom to one in the χ^2^ null distribution and so reduces loss of power through larger degrees of freedom of the previous tests. Unfortunately it is also very unlikely to encounter the situation where one has pooled a set of variants of the same effect size.

The characteristics of these new tests are determined in the analysis of the Results section and placed into context with known results about the case–control score tests.

### Rare Variant Tests

Rare variants [for our purposes, those with minor allele frequency (MAF) less than 1%] that affect disease susceptibility are hard to detect with single SNP association tests. These tests are underpowered unless the sample size is large enough to provide enough subjects with the minor allele present. To overcome the low minor allele counts of rare variants a number of tests have been devised that pool (or collapse) a group of variants into one “super” variant (Morgenthaler & Thilly, 2007; Li & Leal, 2008). This super variant will have a higher minor allele count leading to increased power for detection. However, these collapsing methods introduce their own limitations. Firstly, they only give an indication that at least one of the group is associated with disease susceptibility. Secondly, the selection of SNPs to group together may reduce or nullify the power gained by the increased allele frequency. Power is reduced through increased noise by including noncausal SNPs in the group and through cancellation of effects by including SNPs that have positive associations and SNPs with negative associations. The next generation of rare variant tests attempt to address these issues. Three of the most recent and popular tests are the SKAT test (Wu et al., 2011), kernel-based adaptive cluster test (KBAC; Lui & Leal, 2010) and C-alpha test (Neale et al., 2011).

The C-alpha test utilises the variance of the occurrence of variants within the group. This allows for both protective and risk variants, i.e. causal variants with positive and negative disease association within the group. The test statistic is given by the normalised sum of observed variances assuming a binomial model. Explicitly, let

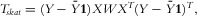
be the (binomial) probability of seeing *a* observations in the cases out of *n* occurrences in both cases and controls with a probability *p*_0_ of each observation being a case and

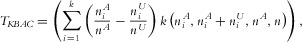
be its variance. The test statistic is

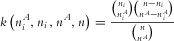
where 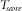 and 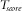 are entries in *A* and *N*, respectively, with 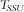 is the number of SNPs with *i* observations. The significance is assessed using a one-tailed standard normal distribution.

The SKAT derives a variance component score statistic from the logistic regression model as

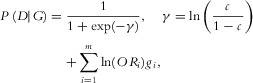
where *W* is a diagonal weight matrix. Under the null hypothesis, the 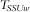 statistic follows a mixture of χ^2^ distributions. The weights 
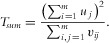
, that lie on the diagonal of *W*, are chosen to place more or less emphasis in the analysis on each of the *m* variants. It is recommended, in rare variant analysis, to define the weights from a beta distribution by 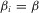 with 
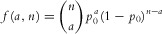
 and 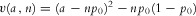 where 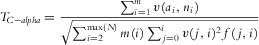 are the observed MAFs at each variant. Wu et al. (2011) use 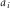 and 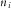 that places greater emphasis on the rare variants and less on common variants. Taking 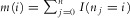 is a special case that is asymptotically equivalent to the C-alpha and SSU tests.

The final rare variant test that we examine is the KBAC test. This test performs a joint comparison of the frequencies of the genotypes that appear in the genetic data between the cases and controls. The first step is to count the number of occurrences of each genotype in the cases and controls. The genotype for each subject is represented by a row in *X*. Let the null genotype be a length *m* row vector of 0s. This equates to a subject having two copies of the major allele at every variant. Let us assume that there are *k* different nonnull genotypes in our data *X*. We let 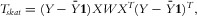 be the counts of each genotype in the cases and 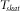 be the counts of each genotype in the controls. The KBAC statistic is

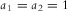
where 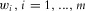 is the total number of affected cases, 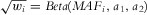 is the total number of unaffected controls and the kernel function *k* is chosen to assign weights according to the size of the data. For small data an hyper-geometric kernel is most appropriate with

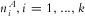
An alternative for small *n* is the marginal binomial kernel and for large *n* is the asymptotic normal kernel (Lui & Leal, 2010). As asymptotic distributions of the statistic under the null are not available *P*-values are obtained empirically via permutation procedures.

It is worth noting that, although not derived as such, the KBAC statistic amounts to a signed difference in genotype frequencies between cases and controls, and is greatest when genotypes are common in cases and rare in controls. Therefore, it is expected to be most powerful when variants are rare in the general population and have effects in the direction of risk, and this has been borne out by simulation studies (Lui & Leal, 2010; Basu & Pan, 2011).

### Family Data and Transformations

Family data in genotyping studies come in many different forms, from parent–child trios to extended pedigrees with many affected relatives. There may even be mixtures of many different pedigree structures. In this study, we choose three basic family structures to demonstrate the key properties of the rare variant association tests.

The first is the parent–child trio, composed of two parents of unknown affection status and a single affected offspring. This is the most common unit of a family-based study design. The second is an affected sib-pair (ASP), consisting of two affected full siblings and their two parents. There, any causal alleles will be enriched over those found in a trio (Risch & Teng, 1998). This structure may cause independence issues due to the nonindependence of genetic information in related cases. An ASP is decomposable into two trios, with the parents appearing in both. The last family structure we term an enriched trio. We use only one trio from an ASP, i.e. we ignore one of the affected siblings. Any causal alleles will be enriched as in an ASP (Risch & Teng, 1998) and there are no independence issues to contend with. We regard this structure as an example of a family-based design in which cases are selected for a family history of disease. Similar designs in the case–control setting have been shown to offer improved power (Antoniou & Easton, 2003; Dudbridge et al., 2012).

Throughout we assume that we have family-based genetic data to begin with. We now describe two ways to transform family-based data into case–control format, to which existing case–control statistical tests may be applied. The data resulting from these transformations are called PCC and UCC. The advantage of having case–control data is that a wider range of tests are available, specifically in the section named Rare Variant Tests.

In the first of these transformations, we utilise the transmission information in each trio to create controls. In each trio a new pseudo-subject, created from the untransmitted alleles of the parents, is taken to be a control. Combining these pseudo-controls with the affected offspring (the cases) creates a data set containing an equal number of cases and controls. We create only one of the three possible pseudo-controls per trio, which is the one based on the two untransmitted alleles, as this corresponds to the unmatched case–control design assumed by the tests we consider (Cordell et al., 2004). The second transformation uses the affected offspring as cases, and samples unrelated controls from the population. Combining these unrelated controls with the cases creates the UCC data set. The question of how these controls are gathered will be addressed in the Discussion section. We apply these two transformations in turn to the same initial family-based data. This reuse of the cases for PCC/UCC formats (and parental data for PCC format) has the effect of mimicking the choices available to real-world data.

In both transformations the new case–control data sets may violate independence assumptions in the derivations of the statistical tests. We address this, when it occurs, by applying a clustering algorithm. In the scenarios defined below with families containing multiple affected offspring then the affected offspring contribute multiple cases to the PCC and UCC data sets. These cases are not independent. For example, two affected siblings are expected to share at least 50% of their genetic make-up. This dependence can lead to underpowered tests or inflated type I error rates. The clustering algorithm we apply sums the count of minor alleles at each variant in related cases to create one case and the same for their matched controls. This reduces the number of cases and controls by a factor equal to the cases in each pedigree (a factor of 2 for ASPs) but maintains independence of the new samples enabling the case–control tests to be applied successfully. Of course this will affect the power, as we will see in the Results section. This approach is equivalent to calculating U and V in the New Family-Based Score Tests section using families as the sampling unit rather than individuals.

### Scenarios

To assess the performance of the various combinations of family structures, data transformations and statistical tests we perform a simulation study. We use simulated data, the transformed data and familial relationships as inputs to each of the statistical tests defined above. We repeat these simulation and statistics steps 1000 times. The power and type I error for each statistical test is determined by the proportion of times that the statistic reports an association at 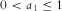.

The overall process is given by the flowchart in Figure S3. When simulating subjects we must determine their affection status. To do this, we employ a logistic model. In the logistic model the chance of being affected by the disease for any given genotype is determined from the following equations:
(1)
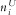
12where 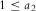 is the probability of a subject being affected with the disease given the subject multi-variant genotype of *G*, *c* is a background chance of being affected for a subject with no minor alleles, 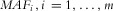 is the effect size of variant *i* and 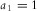 is the number of minor alleles at the *i*th variant. The background chance of being affected with a disease represents the probability of affection from unmeasured factors such as other causal SNPs and environmental factors.

Firstly the six family-based tests (the single SNP TDT and the five new family-based score tests) are applied to the original family data. Next, the nine case–control tests (single SNP association, five score tests, SKAT, KBAC and C-alpha) are applied to the PCC format genetic data. Finally, the same nine tests are applied to the UCC data. These tests are summarised in Table S1. The nonindependence of cases when derived from ASP data, as discussed above (in the Family Data and Transformations section), led to four tests being inappropriate in the UCC setting for ASP derived data. The type I error rates results are presented in the Results section. For the score tests on UCC/ASP data clustering was used. In addition, the asymptotics of the C-alpha test failed due to the low MAF so permutation testing was used and this test is denoted Cα-P.

The parameter space for the simulations includes the familial structure, the number of variants, the MAF at each variant and the number and effect size of any causal variants. To fully explore this parameter space we take a systematic approach to the choices of these simulation parameters. We firstly see how each test performs under the null scenario of no association with all OR equal to 1 for each familial structure. Next, we define three baseline scenarios that provide a starting point for our analysis into the trio, ASP and enriched trio family structures.

The baseline scenarios all contain 10 variants with MAF of 0.5% and a baseline prevalence of 1% [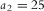 in the logistic model, Eq. (1)], see Table S2. Four of these variants are causal variants, i.e. any minor alleles occurring at any of these four variants confer increase in the chance of being affected by the disease. We choose the OR to be equal for each of the four causal variants and to be set at a level that causes the most powerful test to have a power of approximately 90%. This last condition ensures that we are receiving a clear signal in the sense that were the OR too high then many tests may give (or close to) 100% power or if the OR were too low then the signal would be indistinguishable from the false positives. The OR for trios, ASPs and enriched trios in their baseline scenarios are 2.00, 1.72 and 1.54 that give disease prevalences of 1.21%, 1.15% and 1.11%, respectively.

We then examine perturbations from these baseline parameters. In the first set of scenarios we change the number of causal variants from one to ten. We alter the OR at the same time to maintain approximately 90% power for the most powerful test. This set of scenarios alters the proportion of causal variants within a fixed total number of variants. The next set of scenarios alters the proportion of causal variants by keeping the baseline four causal variants and increasing the total number of variants. For the following two sets of scenarios we fix the number of causal variants at one and four, out of ten, and vary the effect size with the OR ranging from 1.0 to 3.0. The fifth and sixth sets of perturbations on the simulation parameters examine the effects of having nonequal OR causal effect sizes. In the fifth, we include protective variants and in the sixth we fluctuate the OR by up to ±20%. In the seventh, we investigate population stratification by simulating cases from two populations with MAF and OR given in Table S2. Finally, we examine the effect of reducing the sample size. In all the previous scenarios, we have kept the number of cases at 1200; in this set of scenarios we test subsets of size 100 to 1100 for changes in power.

To perform the simulations we use the *dwarf* tool (Preston, 2013). This tool combines a powerful simulator with the ability to call *R* scripts (Ihaka & Gentleman, 1996) and run inbuilt statistical tests. The variants are independently simulated and, as in (Basu & Pan, 2011; Pan, 2009) and others, the conclusions are robust to linkage disequilibrium structure.

## Results

We begin by presenting the type I error rates for each statistical test with the null simulation parameters using a significance level of 5% (see Table 1).

With only one exception for a Cα-P test, the trio and enriched trio false positive rates are all within a strict bound (⩽5.5%) and relaxing this bound does not alter the conclusions below. The single SNP tests (TDT, association and UminP) are shown to be consistently conservative (⩽4%). We found the analytic *P*-value calculation for the C-alpha test to be inaccurate due to the low MAFs encountered in these simulations, hence our use of the permutation method when testing this statistic to correct this problem. The tests on ASP family data are more interesting. All error rates for the UCC data derived from the ASP family data are over 8% deeming them all inappropriate for general use. By using the clustering algorithm on the score tests (see the section named Family Data and Transformations) their type I error rates drop to those presented in Table 1. Three of these error rates are still high but the SSU and sum tests are acceptable.

**Table 1:** The false positive (type I) error rates (in %) for all statistical tests for each family structure (trios, ASPs and enriched trios). Within each family structure there are 24 statistical tests over the three data formats (family, PCC and UCC). Those in bold indicate an error rate of over 5.5%

	Trios			ASPs			Enriched Trios		
	Fam	PCC	UCC	Fam	PCC	UCC	Fam	PCC	UCC
TDT	3.6	–	–	4.9	–	–	3.8	–	–
Assoc.	–	3.6	4.8	–	4.8	**11.0**		3.7	3.9
UminP	3.6	3.8	5.0	5.0	4.8	**6.0**	3.8	3.8	4.4
SSU	4.0	4.1	5.4	5.0	4.9	4.6	5.3	5.3	4.9
Cα-P	–	4.6	**5.8**	–	**5.6**	**16.0**	–	5.5	5.5
SKAT	–	4.2	5.2	–	4.9	**14.8**	–	5.4	5.3
SSUw	4.6	4.5	5.1	5.2	5.1	**6.1**	4.8	4.9	5.4
Score	4.9	4.7	5.1	5.4	5.0	**6.1**	5.5	4.9	5.1
Sum	4.4	4.6	4.6	**6.4**	**6.2**	5.0	4.3	4.3	5.0
KBAC	–	5.5	5.2	–	5.4	**9.8**	–	4.7	4.7

Recall that the baseline scenario consisted of 10 variants with MAF of 0.5% and four causal variants of equal OR dependent on the pedigree structure (2.0 for trios, 1.72 for ASPs and 1.54 for enriched trios). The power estimates for these scenarios are given in Table 2.

**Table 2:** All power results (in %) for the baseline scenarios. The optimal results for three family structures are given in bold and dashes represent tests that failed to maintain a low type I error or are not applicable for that data set. The results are grouped according to correlations between statistical tests, see main text for further details

	Trios			ASPs			Enriched trios		
	Fam	PCC	UCC	Fam	PCC	UCC	Fam	PCC	UCC
TDT	57	–	–	44	–	–	24	–	–
Assoc	–	57	57	–	44	–	–	24	53
UminP	56	58	58	43	45	58	24	24	53
SSU	83	84	85	73	74	**91**	45	45	79
Cα-P	–	84	85	–	75	–	–	46	80
SKAT	–	83	84	–	72	–	–	43	78
SSUw	75	76	78	62	63	82	35	35	73
Score	76	76	78	63	63	81	36	35	72
Sum	78	78	78	72	71	86	45	45	76
KBAC	–	91	**91**	–	85	–	–	62	**89**

The family-based statistical tests and the PCC statistical tests give very closely the same power for every test in each of the family structures (comparing columns 1 and 2, 4 and 5, 7 and 8, Table 2). Furthermore, so do the statistical tests on UCC data derived from trios with the other statistical tests for trios (comparing columns 1, 2 and 3). For the UCC statistical tests we see an increase in power over the equivalent family and PCC tests for tests on ASP (column 6) and enriched trios data (column 9). The increase for ASP data is tempered by the high type I errors seen above.

The table is split into groups. These divide the statistical tests into groups that are theoretically closely related and/or have a large correlation in their power. These relationships are maintained throughout the analyses. Figure S4 demonstrates this for these five groups over the first three sets of scenarios: changing number of causal variants and increasing OR for one and four causal variants. In the right-hand two columns of panels in Figure S4 (for ASP and enriched trio results) we see confirmation of the extra power of UCC tests. We also see a consistent but small reduction in power for the SKAT test compared to the SSU and Cα-P. As the SKAT is a parametrisation/generalisation of these other two tests we conclude that it is the parameters that may need adjusting. Any adjustment may improve the SKAT performance but not by any significant amount to warrant differing conclusions throughout our work.

We use these relationships to clarify the presentation of our results in the subsequent scenarios. From each group and family structure combination we will select one statistic with one data format to be indicative of them all. This gives five statistics to consider for each of the family structures and 15 in total. As there is no significant difference between any of the statistics used in each box for the trio data we select the UminP, SSU, score, sum and KBAC tests with UCC data. This enables our results to be directly compared to those in other work. For enriched trios we make the same selections. The tests on UCC data have a large increase in power throughout.

The results for ASP derived data are not so clear cut. The increase in power when using UCC data is, as for the enriched trios, pronounced but this is at the expense of potentially higher type I errors. We make the same choices as for the trio and enriched trio settings with UminP, SSU, score and sum tests with UCC data and use the KBAC test with PCC as well. The SSUw and score tests that have high type I errors do not perform as well as the SSU and sum tests that have acceptable type I errors. At no point in the analyses below do tests with type I error greater than 5.5% perform optimally.

**Figure 1:**
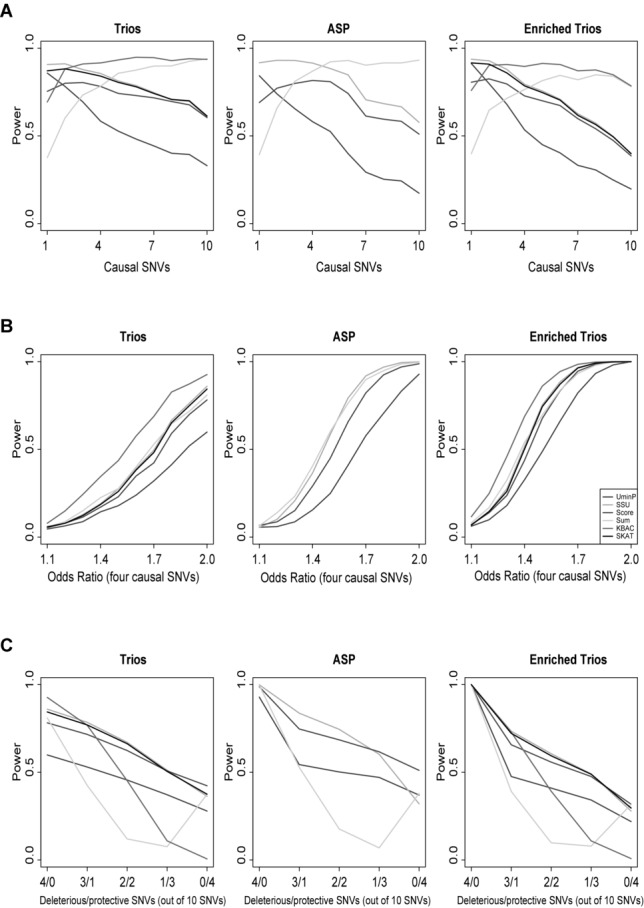
Results for three simulated scenarios. The power is presented in each case with the results for trio derived data on the left, ASP derived data in the middle and enriched trios on the right. (A) The number of causal variants increases from 1 to 10 and the OR is varied to maintain an approximate 90% highest absolute power. (B) For four causal variants we jointly vary their ORs from 1 to 2. (C) While keeping four causal variants we change the direction of the effect of an increasing number of them, from four causal and no protective variants (the baseline scenatio, left) to four protective and no casual variants (right).

Figure 1A presents the relative power of each statistical test as the number of causal variants increases from 1 to 10. The SSU test performs well at low numbers of causal variants. As the proportion of causal variants increase then the KBAC and sum tests come to the fore. The KBAC test is best for trio and enriched trio data at higher numbers of causal variants. It does not perform as well as the sum test for ASP data.

In the second and third set of scenarios, Figures 1B and S1A, we fix the number of causal variants at one and four, respectively, and vary the effect size (OR). The extra sensitivity to low effect sizes in enriched families is clearly evident comparing results for trio data (left panel) to the other results. For a single causal variant the SSU group of tests outperform all other tests including, surprisingly, the single SNP tests with multiple test correction. When there are multiple causal variants the KBAC test has a power increase over the other tests for trio and enriched trio data. The sum and SSU tests are optimal for ASP data and most noticeably the KBAC loses power.

In these results, the SSU test shows optimal power throughout unless the proportion of causal variants is over 20%. The KBAC test demonstrates greater power when the number of causal variants rises above this level but is penalised in the ASP tests. This lack of power has two potential sources: the lack of independence in the cases and the lack of relative genotypic variation found with pseudo-controls.

As we increase the total number of variants in our data, keeping only four causal, we observe in Figure S1B a similar result to Figure 1A. As the proportion of causal variants drops below 20% the SSU test outperforms the KBAC test in trio and enriched trio data. In Figure 1C, where we have replaced an increasing number of the four causal variants with protective ones of equal but opposite protective effect size, the SSU test outperforms the KBAC again. This is to be expected as the SSU is a variance based test, i.e. only the effect deviation contributes irrespective of direction. The sum test, that performs noticably worse, and the KBAC test are not designed to take these variants into account and this reduces the power when they are present.

The fluctuation tests involving perturbations of the effect sizes showed no deviation from the main results and are omitted.

In Table S3 the type I error rates for a stratified population lead to the same conclusions as the baseline tests (c.f. Table 1), namely that the error rates for UCC data derived from the ASP families are too high and that the single SNP tests are consistently conservative. We again employed the permutation method for the C-alpha test and clustering for the score tests for *P*-value calculations. The stratified populations, that are stratified by MAF, OR and baseline prevalence, give the same optimal tests as the baseline scenarios. For all family data using an UCC format is best, with the KBAC test optimal for independent cases (trios and enriched trios) and the SSU test for ASP family data.

An important consideration when choosing appropriate study designs and statistical tests to apply is the amount of data available. In Figure S1C, the power of each statistical test is presented for a subset of the 1000 cases used in each of the baseline scenarios. The power drops off almost linearly in each case. The order of tests by performance in the main results holds, with the exceptions that the sum test outperforms the SSU test at less than 500 cases and the KBAC test for ASP PCC data overtakes both the SSU and sum tests.

**Figure 2:**
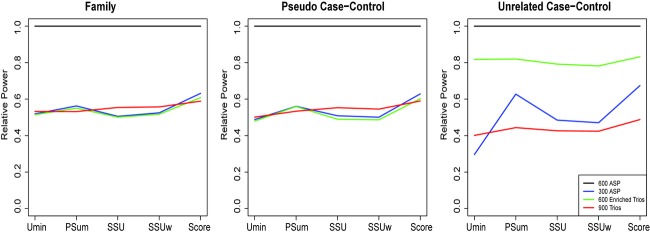
We show the relative power of each of the five score tests to the power of the score tests from data derived from 600 ASPs (1200 cases, black). Results are presented from data derived from 300 ASPs (600 cases, blue), 600 enriched trios (600 cases, green) and 900 trios (900 cases, red). The family-based tests are on the left, the pseudo-case–controls in the middle and unrelated-case–controls on the right.

In Figure 2 we present our key result. This figure presents the relative power of the five score tests for family (left), PCC (middle) and UCC (right). While keeping all other parameters equal (MAFs, ORs, number of causal variants, etc.) the results for simulations derived from 600 ASPs (blue, 1200 cases), 300 ASPs (red, 600 cases), 600 enriched trios (magenta, 600 cases) and 900 trios (black, 900 cases) are displayed. This demonstrates the amount of information in the cases relative to the different test types (transmission or frequency) and different control types (pseudo or unrelated). We clearly see that 600 enhanced cases (from ASPs or enriched trios) are the equivalent of 900 normal cases (from trios) for statistical tests on family-based and PCC data. When unrelated controls are introduced, the results for 600 ASPs and 900 trios remain (albeit with greater variation) at about half of the power of the 1200 ASPs results. For the same number of cases (600), the increase in power in the results for 600 enriched trios data over the results for 300 ASPs data is clear.

## Discussion

Family-based designs have had appeal for detecting association of rare variants because such variants are more common within families with multiple affected subjects. Here, we have compared some basic but key types of family-based design to case–control studies with the same number of cases, using a range of current methods for detecting rare variants. We demonstrate unequivocally that combining cases from enriched families with unrelated controls gives the greatest power to detect rare variants. In particular, when cases with a family history are used, it is more powerful to study these cases in a case–control rather than a case–parent trio or ASP design. In contrast, when unselected cases are used there are no differences in power between family-based and case–control designs.

Figure 2 illustrates this most clearly. In the left two panels 600 cases from ASPs (300 families), 600 cases from enriched trios (600 families) and 900 cases from trios (900 families) are clearly equally powered for both family and PCC statistical tests. When unrelated controls are introduced there is a significant increase in power for the statistical tests when applied to enriched trios. As enriched trios are a typical example of a strong family history of affection we conclude that, for a fixed number of cases, taking one case per pedigree and having more pedigrees is a more powerful design than taking multiple affected individuals from fewer pedigrees.

It may seem surprising that the case–control design has a significant increase in power over the family-based design when the cases have a family history, but not otherwise. The reason is that, under multiplex ascertainment, pseudo-controls constructed from untransmitted haplotypes are not typical of the general population (Thomson, 1995; Cordell, 2004). Therefore the parallel between family-based designs and case–control studies no longer holds, and we have shown that the case–control design is more powerful. While this result holds for both common and rare variants, the presence of a family history is particularly relevant for studies of rare variation as their effects are more likely to be strong and thus to account for that history (Maher, 2008).

Transmission statistics calculated though specific family-based formulae or by using pseudo-controls with case–control methods are shown to have equivalent power. As there are many well-researched and designed case–control statistics to test for rare variant disease associations, the PCC data format provides a larger set of tests to apply than for the original family-based format. For example, while we could easily adapt the SSU (Basu & Pan, 2011) to transmission statistics, the KBAC (Lui & Leal, 2010) is more easily applied to cases and pseudo-controls.

When it comes to selecting the most appropriate statistical test then two groups of tests stand out. The SSU group (including SKAT and C-alpha) and KBAC statistics consistently perform the strongest over the battery of scenarios applied. The equivalencies seen in our results and in analytical work (Basu & Pan, 2011; Wu et al., 2011), and the subsequent groupings we defined (Tables 1 and 2; Fig. S4), conform with expected relationships (e.g. the single SNP tests: TDT, association and UminP are equivalent).

The KBAC test performs the best when there is a high proportion of causal variants (see Figs 1A, B and S1B). Its superiority is clear with 40% causal variants (see Fig.1B). Its suboptimal performance with only one causal variant is shown in Figure S1A. The KBAC test also shows greater relative power at low sample numbers, albeit with an absolute power of less than 50% (see Fig. S1D).

The central unknown of real-world data analysis is the causality of any variant. Therefore, with large GWAS-type studies, is it realistic to expect over 20% of rare variants in a pool to be measureably causal? If the answer to this question is yes then the KBAC test is most suitable. In most studies this assumption cannot be made and the SSU group of statistical tests comes to the fore.

The SSU group consists of the SSU, SKAT and C-alpha tests. These are variance based statistics that perform the best up to 40% causal variants (see Fig.1A) and especially at low proportions (see Fig. S1E). They incorporate protective variants (unlike KBAC; Fig.1C) and maintain strong relative performance at low sample sizes (see Fig. S1F). They also (with clustering) perform the strongest when multiple affected individuals from one pedigree are included in the data set. The SKAT can be considered a generalisation of the other two tests via its parameterisation. The recommended parameters for rare variant testing with SKAT did not outperform the basic tests in our scenarios, so may require further investigation.

Our results with a single causal variant are noteworthy (see Figs S1A and S4). We show the equivalence of TDT, association and UminP statistics for trios and UCC. These contradict the assertions of Laird & Lange (Laird & Lange, 2006), who claimed that trios have greater power than case–controls for low penetrance diseases with rare variants, in contrast to analytic results (Risch & Teng, 1998). Furthermore, we show that the SSU group of tests outperform the single SNP tests with multiple test correction when there is only a single causal variant. This result, which is surprising, does not apply to common variants, and further motivates the use of multivariate statistics such as SSU and SKAT with rare variant data.

In the baseline scenario (Table 2) and the scenarios with varying numbers of causal variants we manipulated the effect size of the causal variants to maintain the optimal power at approximately 90%. The effect sizes used are presented in Figure S2. The required OR to give this optimal power obviously reduces as the number of causal variants increases. The effect size is also much smaller for enriched trios than ASPs and for ASPs than trios. This indicates the greater sensitivity of tests applied to enriched family data. The jump from trios to enriched families (ASPs and enriched trios) can be attributed in large part to the increase in MAF of causal variants in the cases. Cases in ASPs and enriched trios have the same MAF for causal variants but enriched trios show a greater sensitivity. This is due to the increase in power due to the independence of samples. In the ASP and larger pedigree settings nonindependence and the use of a clustering algorithm inhibits power and therefore sensitivity.

We have presented results that indicate future studies should use unrelated controls with cases but what is the implication for existing family-based data? We have shown that when cases are selected from families with multiple affected individuals, it is more powerful to analyse them with unrelated controls than by using transmissions within their families, so a solution might be to identify previously genotyped controls from a matching population and discard the transmission information in the family. Using a statistical test that can incorporate principal components to control for population stratification and admixture, like SKAT, would provide a potential solution. Of course, any study could be improved in this way by adding repository control genotypes, so a more considered view is that the reduced power of such families could be alleviated by combining their transmission data, via pseudo-controls, with repository control genotypes.

We have just touched upon an important point. Family-based statistics, such as those presented here and elsewhere (Lake et al., 2000), have an important property due to using transmission rates. The transmission rate is independent of the underlying allele frequency. The transmission from a parent to a child is also independent of all other transmissions. Thus, family-based statistics control for population stratification and admixture inherently in the information they utilise for their statistics. We have demonstrated above that population stratification does not inflate type I errors, at least in these scenarios. The tests aggregate across multiple rare SNPs and combining SNPs with different allele frequencies from different population strata may give an alterative to family- and transmission-based tests to control for population stratification.

The genotypes of related individuals are not independent therefore those case–control studies that contain related individuals have to contend with nonindependent data as well. These problems are manageable in the case–control setting. As mentioned above, using principle component analysis can control for population stratification and clustering algorithms remove any independence issues when applying case–control statistics to multiple affected individuals in a family.

In conclusion, the use of enriched families is recommended for detecting rare variant disease association, provided this is within a case–control rather than a family-based design. Selecting unrelated rather than family-based controls greatly increases the power of statistical tests and their sensitivity to variants with smaller effect sizes. When cases are not selected for a family history, we found no advantage to using family-based designs compared to case–control studies and the usual concerns of population stratification (Mathieson & McVean, 2012) may be able to be alleviated due to the aggregate nature of the tests. With little prior knowledge of the abundance of causal and protective variants in a pool the SSU group of statistical tests, which includes C-alpha and SKAT, offer the most reliable and informative tests to use for analysis.

## Supporting Information

**Table S1**List of statistical tests. Notes: 1. Not used for ASP as type I error too high. 2. New family-based tests. 3. Clustering for ASP. 4. No family test available. **Table S2**Parameters for population simulation. The most scenarios use the baseline parameters of four causal SNPs and six noncausal all with 0.5% MAF and OR based on the family data. For the stratified tests two equally sized populations with varying MAF, OR and baseline prevalence are used. **Table S3**The false positive (type I) error rates (in %) on a stratified population for all statistical tests for each family structure (trios, ASPs and enriched trios). Within each family structure there are 24 statistical tests over the three data formats (family, PCC and UCC). Those in bold indicate an error rate of over 5.5%. **Table S4**All power results (in %) for the stratified populations. The optimal results for three family structures are given in bold and dashes represent tests that failed to maintain a low type I error or are not applicable for that data set. The results are grouped according to correlations between statistical tests, see main text for further details.

**Figure S1**The results for three scenarios: varying OR for a single causal variant, increasing the noise (noncausal variants) and the sample size. (A) For one causal variant we vary its OR from 1 to 3. (B) The total number of variants in these scenarios rises from 10 to 50 with four causal in each case. (C) These scenarios demonstrate the effect of changing the sample size, from 100 to 1200 cases. **Figure S2**The OR required to give approximately 90% power for the optimal test with increasing number of causal variants out of 10. We see greater sensitivity for enriched trio data (red) than either trio data (blue) or ASP data (green). **Figure S3**A flowchart of analysis for each scenario. We first select the inputs: pedigree structure, number of families, number of variants, MAF profile (MAF for each variants) and OR profile (OR for each variants). Then we repeat this process 1000 times to generate 1000 *P*-values for each statistical test. The proportion below  gives the power for each combination of data, format and test. **Figure S4**The results for the first three sets of scenarios split by correlated group. Each panel consists of three sets of graph, the left being increasing number of causal variants, the middle being increasing OR with 1 causal variant and the right being increasing OR with four causal variants. Each column of panels relates to a data format: trios, ASPs and enriched trios. We see strong correlation in each panel and the increase in power experienced by UCC data in the second and third columns.
