# Supplementary material for: Utilising Family-Based Designs for Detecting Rare Variant Disease Associations
Source: Ann Hum Genet. 2014 Jan 1;78(2):129–40. doi: 10.1111/ahg.12051 (PMC4292528; doi:10.1111/ahg.12051)
Supplement: Supplementary file 1 — Table S1List of statistical tests. Notes: 1. Not used for ASP as type I error too high. 2. New family-based tests. 3. Clustering for ASP. 4. No family test available. Table S2Parameters for population simulation. The most scenarios use the baseline parameters of four causal SNPs and six noncausal all with 0.5% MAF and OR based on the family data. For the stratified tests two equally sized populations with varying MAF, OR and baseline prevalence are used. Table S3The false positive (type I) error rates (in %) on a stratified population for all statistical tests for each family structure (trios, ASPs and enriched trios). Within each family structure there are 24 statistical tests over the three data formats (family, PCC and UCC). Those in bold indicate an error rate of over 5.5%. Table S4All power results (in %) for the stratified populations. The optimal results for three family structures are given in bold and dashes represent tests that failed to maintain a low type I error or are not applicable for that data set. The results are grouped according to correlations between statistical tests, see main text for further details. [file ahg0078-0129-sd1.pdf]

SUPPLEMENTARY INFORMATION

Test	Family	PCC	UCC
TDT	✓	✗	✗
Assoc	✗	✓	✓[1]
UminP	✓[2]	✓	✓[3]
SSU	✓[2]	✓	✓[3]
SSUw	✓[2]	✓	✓[3]
Score	✓[2]	✓	✓[3]
Sum	✓[2]	✓	✓[3]
Cα-P	✗[4]	✓	✓[1]
KBAC	✗[4]	✓	✓[1]
SKAT	✗[4]	✓	✓[1]

Table S.1: List of statistical tests. Notes: 1. Not used for ASP as Type I error too high. 2. New family-based tests. 3. Clustering for ASP. 4. No family test available.

Population	Baseline				Stratified 1		Stratified 2	
	MAF	Trios OR	ASP OR	Enriched OR	MAF	OR	MAF	OR
SNP 1	0.5%	2.00	1.72	1.54	0.8%	1.72	0.2%	1.36
SNP 2	0.5%	2.00	1.72	1.54	0.8%	1.72	0.2%	1.36
SNP 3	0.5%	2.00	1.72	1.54	0.2%	1.36	0.8%	1.72
SNP 4	0.5%	2.00	1.72	1.54	0.2%	1.36	0.8%	1.72
SNPs 5-10	0.5%	1	1	1	0.5%	1	0.5%	1
Prevalence	1%				1.2%		0.8%	

Table S.2: Parameters for population simulation. The most scenarios use the baseline parameters of four causal SNPs and six non-causal all with 0.5% MAF and OR based on the family data. For the stratified tests two equally sized populations with varying MAF, OR and baseline prevalence are used.

	Trios			ASPs			Enriched Trios		
	Fam	PCC	UCC	Fam	PCC	UCC	Fam	PCC	UCC
TDT	4.2	-	-	4.2	-	-	3.9	-	-
Assoc.	-	4.2	3.6	-	4.2	**9.3**		3.7	4.1
UminP	4.3	4.4	3.8	4.1	4.1	**5.2**	3.8	3.9	4.3
SSU	5.1	5.2	4.2	3.6	3.6	4.0	3.8	3.7	3.9
$C\alpha$-P	-	5.2	4.4	-	4.2	**14.0**	-	4.0	4.0
SKAT	-	5.3	4.5	-	3.5	**13.5**	-	3.6	3.8
SSUw	5.3	5.3	4.2	3.3	3.2	5.1	3.7	3.5	3.9
Score	5.1	5.2	4.2	3.4	3.3	5.3	4.0	4.0	3.6
Sum	5.2	4.9	4.6	**5.6**	5.3	**5.7**	4.6	4.6	4.3
KBAC	-	5.2	4.9	-	**5.7**	**8.6**	-	5.1	4.7

Table S.3: The false positive (Type I) error rates (in %) on a stratified population for all statistical tests for each family structure (trios, ASPs and enriched trios). Within each family structure there are 24 statistical tests over the three data formats (family, PCC and UCC). Those in bold indicate an error rate of over 5.5%.

	Trios			ASPs			Enriched Trios		
	Fam	PCC	UCC	Fam	PCC	UCC	Fam	PCC	UCC
TDT	24	-	-	29	-	-	30	-	-
Assoc	-	24	22	-	29	-	-	29	66
UminP	25	24	23	30	30	-	29	29	67
SSU	24	34	35	51	51	**73**	50	50	86
Cα-P	-	35	36	-	52	-	-	50	87
SKAT	-	33	35	-	47	-	-	47	85
SSUw	30	30	30	39	39	56	39	38	80
Score	33	30	30	41	40	55	41	39	79
Sum	24	33	33	44	44	-	48	48	77
KBAC	-	49	**50**	-	-		-	63	**92**

Table S.4: All power results (in %) for the stratified populations. The optimal results for three family structures are given in bold and dashes represent tests that failed to maintain a low Type I error or are not applicable for that data set. The results are grouped according to correlations between statistical tests, see main text for further details.
