# Supplementary material for: Utilising Family-Based Designs for Detecting Rare Variant Disease Associations
Source: Ann Hum Genet. 2014 Jan 1;78(2):129–40. doi: 10.1111/ahg.12051 (PMC4292528; doi:10.1111/ahg.12051)
Supplement: Supplementary file 3 — Figure S1The results for three scenarios: varying OR for a single causal variant, increasing the noise (noncausal variants) and the sample size. (A) For one causal variant we vary its OR from 1 to 3. (B) The total number of variants in these scenarios rises from 10 to 50 with four causal in each case. (C) These scenarios demonstrate the effect of changing the sample size, from 100 to 1200 cases. Figure S2The OR required to give approximately 90% power for the optimal test with increasing number of causal variants out of 10. We see greater sensitivity for enriched trio data (red) than either trio data (blue) or ASP data (green). Figure S3A flowchart of analysis for each scenario. We first select the inputs: pedigree structure, number of families, number of variants, MAF profile (MAF for each variants) and OR profile (OR for each variants). Then we repeat this process 1000 times to generate 1000 P-values for each statistical test. The proportion below gives the power for each combination of data, format and test. Figure S4The results for the first three sets of scenarios split by correlated group. Each panel consists of three sets of graph, the left being increasing number of causal variants, the middle being increasing OR with 1 causal variant and the right being increasing OR with four causal variants. Each column of panels relates to a data format: trios, ASPs and enriched trios. We see strong correlation in each panel and the increase in power experienced by UCC data in the second and third columns. [file ahg0078-0129-sd3.pdf]

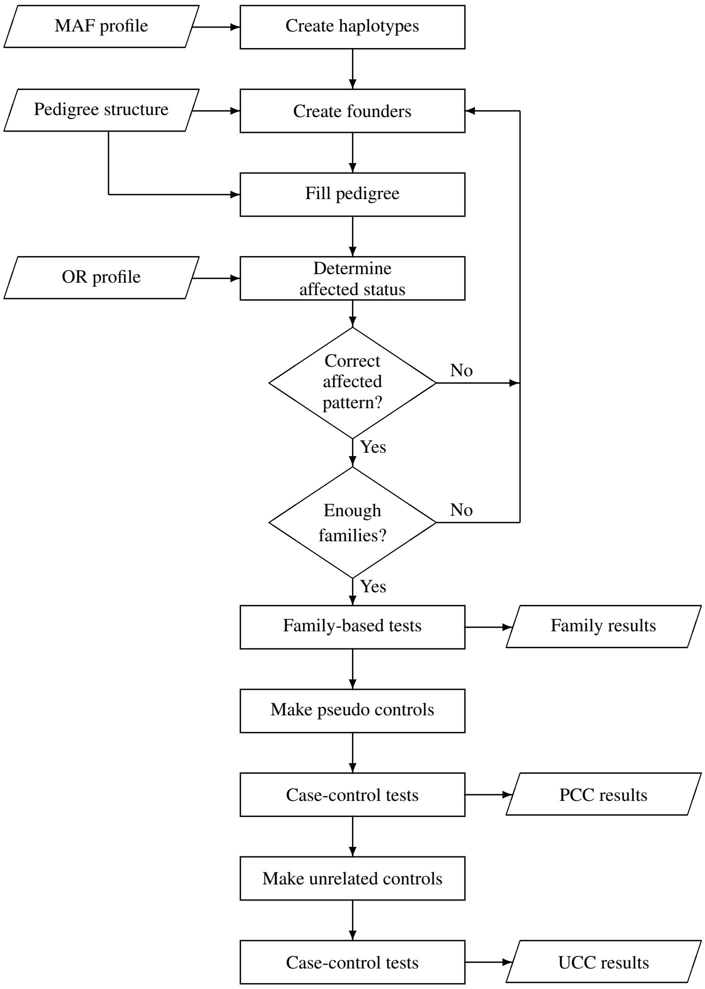

Figure S.3: A flowchart of analysis for each scenario. We first select the inputs: pedigree structure, number of families, number of variants, MAF profile (MAF for each variants) and OR profile (OR for each variants). Then we repeat this process 1000 times to generate 1000 p-values for each statistical test. The proportion below $\alpha = 0.05$ gives the power for each combination of data, format and test.

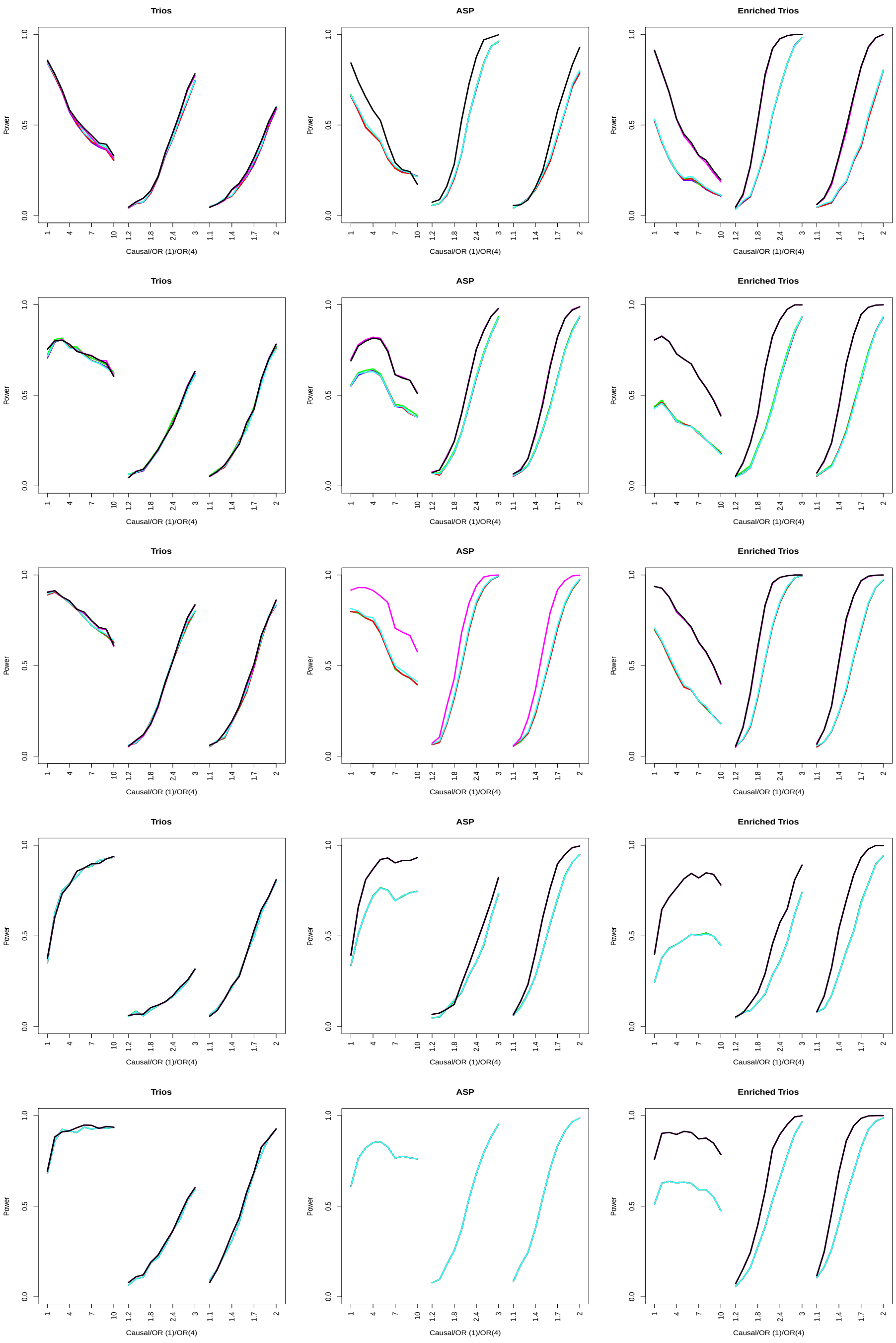

Figure S.4: The results for the first three sets of scenarios split by correlated group. Each panel consists of three sets of graph, the left being increasing number of causal variants, the middle being increasing OR with 1 causal variant and the right being increasing OR with 4 causal variants. Each column of panels relates to a data format: trios, ASPs and enriched trios. We see strong correlation in each panel and the increase in power experienced by UCC data in the second and third columns.

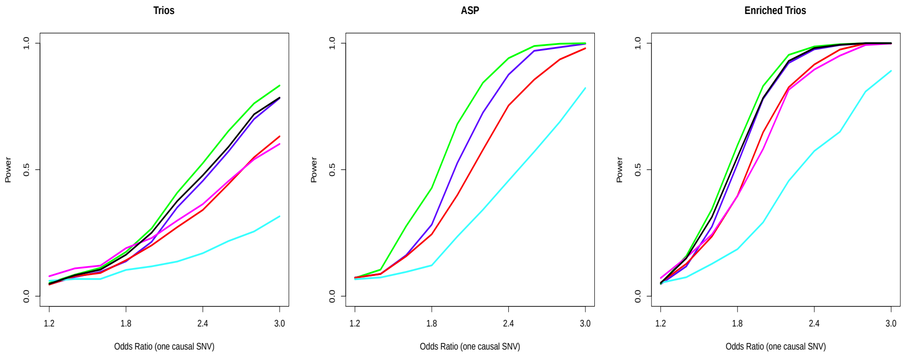

(a) For one causal variant we vary its OR from 1 to 3.

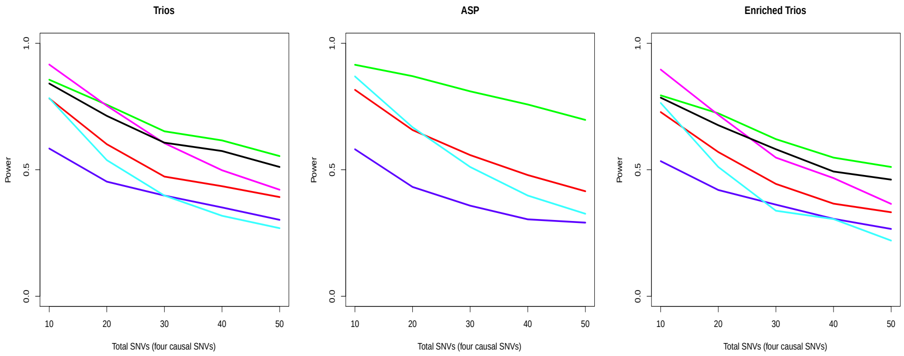

(b) The total number of variants in these scenarios rises from 10 to 50 with four causal in each case.

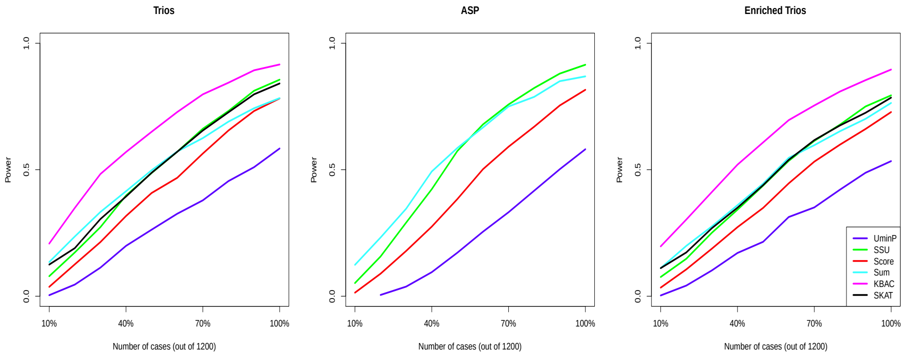

(c) These scenarios demonstrate the effect of changing the sample size, from 100 to 1200 cases.

Figure S.1: The results for three scenarios: varying OR for a single causal variant, increasing the noise (non-causal variants) and the sample size.

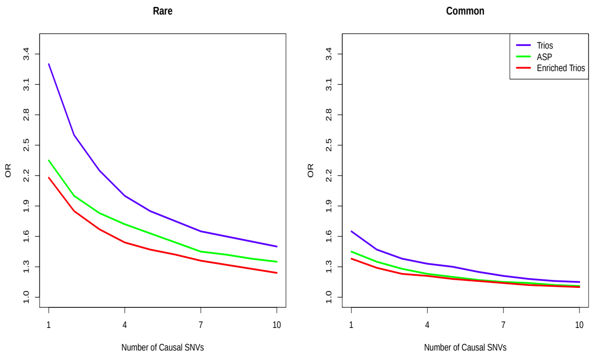

Figure S.2: The OR required to give approximately 90% power for the optimal test with increasing number of causal variants out of ten. We see greater sensitivity for enriched trio data (red) than either trio data (blue) or ASP data (green).
